# Enhanced xylose fermentation and ethanol production by engineered *Saccharomyces cerevisiae* strain

**DOI:** 10.1186/s13568-015-0102-y

**Published:** 2015-02-26

**Authors:** Leonardo de Figueiredo Vilela, Verônica Parente Gomes de Araujo, Raquel de Sousa Paredes, Elba Pinto da Silva Bon, Fernando Araripe Gonçalves Torres, Bianca Cruz Neves, Elis Cristina Araújo Eleutherio

**Affiliations:** Department of Biochemistry, Institute of Chemistry, Federal University of Rio de Janeiro, Rio de Janeiro, Brazil; Department of Cellular Biology, Institute of Biology, University of Brasília, Brasília, DF Brazil

**Keywords:** Evolutionary engineering, Xylose isomerase, Saccharomyces cerevisiae, TAL1, Xylose, Ethanol

## Abstract

We have recently demonstrated that heterologous expression of a bacterial xylose isomerase gene (*xylA*) of *Burkholderia cenocepacia* enabled a laboratorial *Saccharomyces cerevisiae* strain to ferment xylose anaerobically, without xylitol accumulation. However, the recombinant yeast fermented xylose slowly. In this study, an evolutionary engineering strategy was applied to improve xylose fermentation by the *xylA*-expressing yeast strain, which involved sequential batch cultivation on xylose. The resulting yeast strain co-fermented glucose and xylose rapidly and almost simultaneously, exhibiting improved ethanol production and productivity. It was also observed that when cells were grown in a medium containing higher glucose concentrations before being transferred to fermentation medium, higher rates of xylose consumption and ethanol production were obtained, demonstrating that xylose utilization was not regulated by catabolic repression. Results obtained by qPCR demonstrate that the efficiency in xylose fermentation showed by the evolved strain is associated, to the increase in the expression of genes *HXT2* and *TAL1,* which code for a low-affinity hexose transporter and transaldolase, respectively. The ethanol productivity obtained after the introduction of only one genetic modification and the submission to a one-stage process of evolutionary engineering was equivalent to those of strains submitted to extensive metabolic and evolutionary engineering, providing solid basis for future applications of this strategy in industrial strains.

**Electronic supplementary material:**

The online version of this article (doi:10.1186/s13568-015-0102-y) contains supplementary material, which is available to authorized users.

## Introduction

The ethanol production from lignocellulosic biomass is a promising alternative energy source. *Saccharomyces cerevisiae* is the preferred microorganism used to produce ethanol due to its excellent ability to ferment glucose in addition to its high tolerance to ethanol and inhibitors presented in lignocellulosic hydrolysates (Stambuk et al. 2008). However, it is not capable to ferment xylose, present in significant amounts in biomass hydrolysates. In *S. cerevisiae,* xylose is converted into xylulose via two enzymes that use different cofactors, leading to a redox imbalance and, consequently, prevents xylose fermentation. Two main strategies have been commonly applied to solve this problem: the cloning of a xylose reductase and a xylitol dehydrogenase which are linked to the same coenzyme or the cloning of a xylose isomerase which converts xylose directly into its isomer xylulose. Nevertheless, yeasts engineered through that strategy to ferment xylose still do it slowly and accumulate xylitol (Kim et al. 2012). Thus, additional genetic modifications have been carried out as an attempt to increase the specific consumption of xylose as well as the rate and yield of production of ethanol: i) overexpression of the enzymes necessary for the conversion of xylulose into glycolysis intermediates; ii) deletion of the endogenous aldose reductase which converts xylose into xylitol; iii) overexpression of heterologous xylose transporters (Cai et al. 2012). Besides the metabolic engineering approach, evolutionary engineering has been employed to improve the cell performance for ethanol production and to increase the stability of the recombinant strains (Cai et al. 2012). Evolutionary engineering strategies are in fact complementary to metabolic engineering in the search for desired phenotypes through the imposition of one or more selective pressures. Although a great diversity of metabolic engineering and adaptation approaches have been tested to boost xylose fermentation in biomass hydrolysates, yield and productivity of ethanol by genetically engineered *S. cerevisiae* strains are still much lower than those of glucose fermentation.

The simultaneous conversion of xylose and glucose is another bottleneck to the economic ethanol production from biomass hydrolysates (Ha et al. 2011; Eiteman et al. 2008). Yeasts engineered to ferment xylose are not able to consume xylose until glucose is completely exhausted. One possible explanation for this phenomenon is that glucose represses the expression of genes necessary to the catabolism of xylose through Mig1 an important and essential transcription factor for the process of catabolic repression. In the presence of high levels of glucose, Mig1 rapidly moves from the cytoplasm into the nucleus and binds to the promoters of glucose-repressible genes. When the cells are deprived of glucose, Mig1 is transported back to the cytoplasm, releasing glucose repression (Rolland et al. 2002). In an attempt to overcome the inhibitory effect of glucose over the use of xylose, a recent report described an engineered yeast strain, designed to perform intracellular hydrolysis of cellobiose, allowing co-consumption of cellobiose and xylose (Ha et al. 2011). Noteworthy, the sequential use of xylose after glucose depletion could also be attributed to the competition between xylose and glucose during uptake. In *S. cerevisiae*, this pentose is transported by facilitated transport mediated by hexose permeases which transport xylose with very low affinity compared with uptake of glucose (Subtil and Boles 2012). Discovery or engineering of a xylose-specific transporter which is not inhibited by glucose and which shows high affinity and capacity of transport might improve cellular performance to ferment xylose in biomass hydrolysates (Weber et al. 2010).

Recently, we described the functional expression of *Burkholderia cenocepacia* xylose isomerase and its effect on the ability of *S. cerevisiae* to ferment xylose-glucose blends (De Figueiredo Vilela et al. 2013). A major breakthrough, the ethanol yields obtained by the sole heterologous expression of the bacterial enzyme was equivalent to those of strains submitted to extensive metabolic and evolutionary engineering. The recombinant strain did not accumulate xylitol, but it still consumed xylose very slowly compared to glucose, resulting in relatively low ethanol productivities. In the present work, we report an evolutionary engineering approach which promoted an increase on the consumption rate of xylose and ethanol production by the *S. cerevisiae* expressing xylose isomerase from *B. cenocepacia*. Aiming to unravel the changes between the metabolically engineered and the subsequently evolved strain, the expression profile of several genes involved in xylose fermentation was analyzed.

## Methods

### Strain and adaptive evolution experiment

*Saccharomyces cerevisiae* BY4741 (*MATa, his3, leu2, met15, ura3*) expressing xylose isomerase from *Burkholderia cenocepacia* (de Figueiredo Vilela et al. 2013) was used in this study. The adaptive evolution experiments were performed in 500 mL flasks at 28°C/160 rpm filled with 100 mL of YNB (0.7% yeast nitrogen base without amino acids; 0.01% histidine; 0.01% leucine; 0.01% metionine) containing 2% xylose. Initial cell concentration was 0.5 mg/mL (8,3 × 10^6^ UFC/mL). A serial transfer of 50 mg of cells (dry weight) into fresh medium was performed every 24 h. After 40 passages, cells were plated on solid YNB medium. An isolated colony was used in the experiments (Subtil and Boles 2012). Both original (un-evolved) and evolved strains were grown in YNB medium containing different proportions of glucose-xylose (D4%, D3%X1%, D2%X2%, D1%X3%, X4%) until exponential growth phase. Next, they were collected by centrifugation and transferred to fermentation media.

### Fermentation conditions

Fermentation was carried out at 30°C and pH 5.0, in flasks of 50 mL filled with 25 mL of fermentation medium (4.0% Total sugar; 0.4% (NH_4_)_2_SO_4_ and 0.4% KH_2_PO_4_, pH 5) (Zhou et al. 2012). Initial cell concentration was 1.5 mg/mL (2,5 × 10^7^ UFC/mL). Over the time, samples were collected, centrifuged and the cell-free supernatants were used for the determination of glucose and xylose consumption as well as xylitol and ethanol production. The concentrations of glucose, xylose and xylitol were determined by HPLC (Shimadzu) equipped with refractive index detector. The column used for separation was a LiChrospher NH_2_ (Merck). The HPLC apparatus was operated with a mobile phase of 80% acetronitrile and 20% H_2_O at a flow rate of 1.0 mL/min (Ferreira et al. 1997). The ethanol concentration was assessed by dichromate oxidation method with supernatants after distillation (Seo et al. 2009).

### Expression analysis

The un-evolved and evolved strains were cultivated until exponential growth phase in YNB medium containing glucose as carbon source. For enzyme assays, cells were harvested and disrupted with glass beads (diameter 0.45 mm, Sigma Aldrich, U.S.A.). Protein concentration was determined with Stickland assays using bovine serum albumin as a standard (Stickland 1951). Xylose isomerase activity was measured as previously described (de Figueiredo Vilela et al. 2013). Quantitative qPCR was used to measure the expression of *XKS1, RPE1, RKI1, TAL1, TKL1, HXT1, HXT2* and *HXT7. TAF10* (RNA pol II transcription factor activity/transcription initiation and chromatin modification) was used as an endogenous control (Teste et al. 2009). Gene sequences were analyzed using File Builder® 3.1 v2.0 (Applied Biosystems, U.S.A.). TaqMan probes were synthesized by Life Technologies (Applied Biosystems, USA). QuickPrep mRNA Purification Kit (GE Healthcare Life Sciences) was used to extract mRNA from cells harvested in the mid-log phase. Ready to Go RT-PCR Beads (GE Healthcare Life Sciences) was used to for cDNA synthesis. All real time PCR reactions were performed on a Stepone System Real Time PCR cycler (Applied Biosystems, U.S.A.) according to manufacturer’s instructions. The 2 ^–ΔΔCT^ method was used to analyze the relative changes in gene expression in evolved and un-evolved strains (Livak and Schmittgen 2001).

## Results

### Evolutionary engineering promotes high levels of xylose consumption in the presence of glucose

The adaptive evolution experiments were performed with 40 passages in YNB medium and isolated clones were screened. A single clone was than selected for further analyses outstanding results. To investigate the effect of this engineering approach on the capacity of the evolved clone to consume xylose in the presence of glucose, cells were pre-grown on in YNB medium containing different proportions of glucose-xylose until exponential growth phase and then transferred to a fermentation medium containing 2% xylose and 2% glucose. Interestingly, the evolved strain showed a substantially higher xylose consumption rate than the un-evolved strain (around 10-fold higher), as shown in Table 1. Furthermore, the rate of xylose consumption during fermentation shown by cells pre-grown under higher glucose concentrations was more than 80% of the rate of glucose consumption.

**Table 1:** **Specific glucose and xylose consumption rates**

**Growth medium** **containing YNB**	**Specific Consumption (g/L/h) in the fermentation medium (D2%X2%)**									
	**D4%**		**D3%X1%**		**D2%X2%**		**D1%X3%**		**X4%**	
**Strains**	**Glucose**	**Xylose**	**Glucose**	**Xylose**	**Glucose**	**Xylose**	**Glucose**	**Xylose**	**Glucose**	**Xylose**
**Non-adapted**	0.48 ± 0.07	0.08 ± 0.04	0.47 ± 0.02	0.08 ± 0.02	0.47 ± 0.02	0.06 ± 0.04	0.45 ± 0.02	0.06 ± 0.03	0.41 ± 0.01	0.05 ± 0.02
**Adapted**	0.73 ± 0.03	0.60 ± 0.02	0.73 ± 0.07	0.59 ± 0.09	0.73 ± 0.05	0.55 ± 0.03	0.73 ± 0.06	0.55 ± 0.02	0.70 ± 0.09	0.49 ± 0.02

Initially, both original (un-evolved) and evolved on xylose strains were grown in YNB medium containing different proportions of glucose-xylose until mid-log phase of growth and, then, shifted to the fermentation medium containing 2% glucose and 2% xylose. The aliquots were harvested in time of 24 hours, centrifuged and the supernatants were used to determine the concentration of glucose and xylose, which were used to calculate the rate of sugar consumption. The results represent the mean ± standard deviation of at least three independent experiments.

### Evolutionary engineering improves xylose-specific consumption and ethanol productivity

To investigate and compare the fermentative performance of the evolved and un-evolved strains, cells were pre-grown in YNB medium supplemented with 4% glucose until exponential growth phase. Eventually, strains were transferred to a fermentation medium containing glucose and xylose at the same concentration and proportion found in sugar cane hydrolyzates (3.0% glucose plus 1.0% xylose).

**Figure 1:**
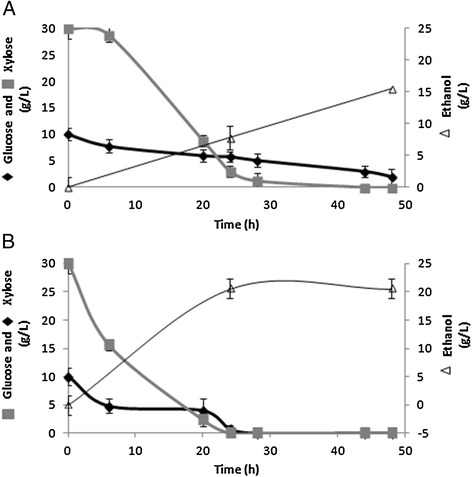
**Sugar consumption and ethanol production by** ***S. cerevisiae.*** Cells of un-evolved **(A)** and evolved strains **(B)** were grown in YNB-medium supplemented with 4% glucose until mid-log phase, collected by centrifugation, washed with distilled water and transferred to fermentation medium containing 3% glucose and 1% xylose. The samples were collected in times of 0, 6, 20, 24, 28, 44, 48 hours and the supernatants were used to determine the concentration of glucose and xylose, in times of 24 and 48 hours the supernatants were used to determine the ethanol concentration. The results represent the mean ± standard deviation of at least three independent experiments.

The evolutionary engineering strategy significantly improved the specific xylose consumption rate, and provided efficient ethanol production from this sugar cane-like xylose-glucose mixture. The ethanol yield and productivity showed by the evolved strain were 13% (0.51 × 0.45 g ethanol/g sugar) and 120% (0.42 × 0.19 g ethanol/g cell/h) higher, respectively, than that of the un-evolved strain (Figure 1A, B). The evolved strain did not show xylitol accumulation, unlike the un-evolved strain (results not shown).

### Analyses of gene expression profiles demonstrate up-regulation of genes for transaldolase and a hexose transporter

**Figure 2:**
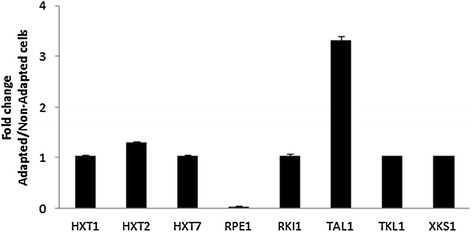
**Gene expression profiles of genes involved with xylose utilization by** ***S. cerevisiae.*** The real-time quantitative (qRT-PCR) was used to detect the mRNA expression level of genes involved with xylose catabolism. Gene expression, calculated as fold change compared to the endogenous control gene *TAF1*0, was determined by qRT-PCR in cells harvested at the middle of log-phase of growth. Fold change between un-evolved and evolved strains was evaluated by the 2 ^– ΔΔCT^ method. All the results were expressed as the mean ± standard deviation of at least three independent experiments.

The un-evolved and evolved strains were grown to exponential growth phase in YNB medium containing glucose as the sole carbon source. Quantitative qRT-PCR was used to measure the expression of genes known to be involved in xylose fermentation (Additional file 1: Table S1). The 2 ^–ΔΔCT^ method was used to analyze the relative changes in gene expression in evolved and un-evolved strains. Xylose isomerase activity was measured but no significant differences in xylose isomerase activity between both strains were observed (data not shown). Results obtained by qRT-PCR (Figure 2) suggest that the efficient xylose fermentation showed by the evolved strain can be attributed, at least in part, to the elevated expression of *TAL1*, which codes for transaldolase*.* The evolved strain presented a 3.0 fold-higher *TAL1* expression than the un-evolved strain. Expression of *HXT2*, that codes for an hexose-transporter was also measured, whilst no important change was observed within the expression of the other target genes.

## Discussion

In this work, an evolutionary engineering approach was applied to select a spontaneous mutant with higher specific xylose consumption rate. For this, the recombinat xylose-fermenting strain expressing xylose isomerase from *B. cenocepacia* was submitted to sequential batch cultivations in YNB-medium supplemented with 2% xylose. According to some authors (Ha et al. 2011), the delay in the xylose consumption caused by glucose is related to the catabolic repression phenomenon. To investigate the effect of this engineering approach on the capacity of cells to consume xylose in the presence of glucose, cells were pre-grown on YNB-media containing different glucose-xylose mixtures and subsequently transferred to a fermentation medium containing 2% xylose and 2% glucose. Similarly to a previous report (Stambuk et al. 2008), our fermentation processes used high yeast cell densities and media poor in nutrients to increase ethanol yield, in detriment of cell growth rate. According to Table 1, the evolved strain showed a substantially higher xylose consumption rate when compared to the un-evolved strain (around 10-fold higher). Furthermore, the rate of xylose consumption by cells pre-grown under higher glucose concentrations was more than 80% of the rate of glucose consumption. Interestingly, the beneficial effect of glucose on xylose consumption was observed in both strains, strongly indicating that xylose metabolism is not subjected to catabolic repression. These results lead to the conclusion that the success of evolutionary engineering should not relate to the selection of unrepressed derivatives, which no longer respond to the presence of glucose, as previously suggested in the literature (Ha et al. 2011).

According to some authors, glucose repression is a significant barrier to successful utilization of mixed sugars in cellulosic hydrolyzates (Ha et al. 2011). In contrast, the present work demonstrated that the xylose specific consumption during fermentation of a glucose-xylose blend was improved when cells were previously grown on glucose, as opposed to xylose alone.

In xylose-grown cells, the glycolytic flux to piruvate is 25 to 30 times lower than in glucose-grown cells (Klimacek et al. 2010). Probably, it is a consequence of the increased expression of genes repressed via Mig1 in the xylose-grown cells (Salusjärvi et al. 2008) as Mig1 is dephosphorylated (a form in which it represses its target genes) only at high glycolytic rates (Elbing et al. 2004b). Confirming this hypothesis, it has been reported that the expression of genes encoding respiratory, tricarboxylic acid (TCA) cycle, and gluconeogenic enzymes is higher during growth on xylose than in glucose repressed cells (Elbing et al. 2004b). Thus, when growing in the presence of xylose, yeast cells switch the mode of metabolism from fermentation to respiratory, leading to a reduction in the glycolytic flux. By reducing the concentration of intracellular piruvate, respiration is favored in detriment to the ethanol production since the Km for the mitochondrial piruvate dehydrogenase alpha is 0.65 mM (Complex 1981) versus 2.29 mM for the piruvate decarboxylase 1, the key enzyme in alcoholic fermentation (Sergienko and Jordan 2001). On the other hand, xylose seems to be sensed by yeast as a non-fermentable carbon source. The presence of this pentose reduces significantly the activity of hexokinase 2 (Hxk2), a crucial regulator of the glucose repression signal in *S. cerevisiae* (Bergdahl et al. 2013). Hxk2 is dephosphorylated on fermentable carbon sources, whereas both phosphorylated and dephosphorylated forms exist on poorly fermentable carbon sources (Randez-Gil et al. 1998). Two of the three phosphorylated forms of Hxk2 are present in cells grown on xylose (Salusjärvi et al. 2008). Therefore, increasing the intracellular concentration of glycolytic intermediates would affect, in turn, the transcription of glycolytic and ethanologenic enzymes. This may provide the metabolic basis to explain why xylose was utilized at a higher rate in fermentation media when cells were pre-grown in the presence of increased concentrations of glucose.

Next, we compared the fermentative performance of the evolved stain to the un-evolved strain. Cells were pre-grown in YNB medium supplemented with 4% glucose until exponential growth phase and then transferred to a fermentation medium containing glucose and xylose at the same concentration and proportion found in sugar cane hydrolyzates (3.0% glucose plus 1.0% xylose). According to Figure 1A and B, the evolutionary engineering strategy improved significantly the xylose specific consumption rate besides the efficiency of ethanol production from this xylose-glucose mixture. The ethanol yield and productivity showed by the evolved strain were 13% (0.51 × 0.45 g ethanol/g sugar) and 120% (0.42 × 0.19 g ethanol/g cell/h) higher, respectively, than that of the un-evolved strain. Furthermore the evolved strain did not show xylitol accumulation, such as occurred in un-evolved strain (results not shown). The ethanol yield and productivity obtained by the strategy used in this work was equivalent, or even better, to those of strains submitted to extensive metabolic and evolutionary engineering. Recent studies described the construction of genetically modified and evolved strains of *S. cerevisiae,* which were able to ferment xylose as a sole carbon with productivities between 0.2 and 0.8 g ethanol/g cells/h (Zhou et al. 2012; Kim et al. 2013; Demeke et al. 2013; Hector et al. 2013). According to Cai and collaborators (Cai et al. 2012), who compared the performance of 30 engineered *S. cerevisiae* strains, the best result was achieved with the recombinant and evolved strain RWB218, carrying the xylose isomerase of the fungus *Piromyces sp*., besides several additional genetic modifications (deletion of *GRE3* and over expression of *XKS1, TAL1, TKL1, RKI1* and *RPE1*). That strain showed ethanol yield of 0.43 g ethanol/g sugar and productivity of 0.2 g ethanol/g cell/h. A similar fermentation performance was achieved by the engineered *S. cerevisiae* strain overexpressing *Piromyces sp. XYLA*, *Pichia stipitis XYL3* and all genes of the non-oxidative pentose phosphate pathway, besides being submitted to a three-stage process of xylose adaptation (Zhou et al. 2012). Although most of the recombinant *S. cerevisiae* strains carrying heterologous xylose reductase-xylose dehydrogenase genes produced considerable amounts of xylitol at a high yield, the strain F106KR, expressing a xylose reductase, which preferred NADPH to NADH and containing several other genetic modifications, showed a high capacity to produce ethanol from high xylose concentrations (Xiong et al. 2011). The yields of F106KR from 100 g/L glucose and 100 g/L xylose in 72 h were 0.42 g ethanol/g and 0.07 g xylitol/g.

Subsequently, the expression of genes known to be involved in xylose fermentation (Additional file 1: Table S1) was analyzed. No significant differences in xylose isomerase activity between both strains were observed (data not shown). Results obtained by qRT-PCR (Figure 2) suggest that the efficient xylose fermentation showed by the evolved strain can be attributed, at least in part, to the elevated expression of *TAL1*, which codes for transaldolase*.* The evolved strain presented a 3.0 fold-higher *TAL1* expression than the un-evolved strain. According to Klimacek and collaborators (Klimacek et al. 2010) the utilization of xylose instead of glucose has several effects on the yeast metabolome that are specific to anaerobic consumption of xylose. For example, the reaction catalyzed by Tal1 is strongly shifted away from its equilibrium, indicating that this reaction is a rate-limiting step for the conversion of xylose into ethanol. Confirming our study that transaldolase has a great influence on xylose utilization, it has been previously demonstrated that the overexpression of the transaldolase from *Pichia stipitis* in *Fusarium oxysporum* significantly improves ethanol production from xylose (Fan et al. 2011).

According to Figure 2, in addition to *TAL1, HXT2* (encoding a low-affinity hexose transporter) was up-regulated significantly in the evolved strain. In the evolved strain, the expression of *HXT2* was at least 30% higher when compared with un-evolved strain. *S. cerevisiae* take up xylose through the family of hexose transporters, which have a much higher affinity for glucose than xylose (Cai et al. 2012). The transporters Hxt1 to Hxt4 plus Hxt6 and Hxt7 are the most important for the uptake of xylose; they display distinct transport capacities and affinities for this pentose (Cai et al. 2012). Among them, Hxt2 has the second highest transport capacity, being able to take up xylose at a rate of 8.74 g/h/g dry weight of cell at high sugar concentrations. Therefore, it should be expected a positive effect on xylose utilization under increased *HXT2* expression, such as occurred in the evolved strain. The expression of the genes which code for hexose transporters depends on the glucose concentration in the medium (Elbing et al. 2004a). In our experiments, cells were pre-grown in YNB-medium containing 4% glucose, a condition which activates *HXT2* expression, which might have greatly improved the xylose utilization.

Zha et al. (2014) conducted a comparative analysis of gene expression of XDH in *S. cerevisiae*, which is NADP^+^ dependent, before and after the evolutionary engineering, Which lead to an increase in the expression of RPE1. This report suggested that the increase in RPE1 expression is due to the greater need for the formation of ribose-5-phosphate, since a sufficient amount of precursor is required when cells are grown on xylose, to maintain the balance between glycolysis and aromatic amino acids and nucleic acids biosynthesis pathways. In conclusion, the results showed in our work confirmed that the reduction on RPE1 expression was observed and could be related to the low need of ribose-5-phosphate by *S. cerevisiae* in fermentation process, with no problem on biomass yield. Therefore maintaining the balance between glycolysis and biosynthetic pathways of aromatic amino acids and nucleic acids was not necessary.

The efficiency of the evolutionary engineering strategy used herein is promising and the fermentation performance of the xylose-evolved recombinant strain was remarkable when compared to previous reports in the literature (Cai et al. 2012; Zhou et al. 2012). However, genetic alterations that occurred during the evolution process remains to be further assessed. Additional studies are necessary to gain insight into the possible mutations which are related to the observed physiology phenotype.

Finally, the evolutionary engineering of our recombinant yeast strain fermented glucose and xylose rapidly and almost simultaneously, showing a substantial improvement in ethanol production and productivity. It was also observed that when cells were grown in a medium containing higher glucose concentration, before being transferred to fermentation medium, higher xylose consumption rates were obtained, demonstrating that xylose utilization was not regulated by catabolic repression. In addition, results obtained by qRT-PCR suggested that the efficiency in xylose fermentation should be attributed to, at least in part, the increasing on *HXT2* and *TAL1* expression.

## Additional file

Additional file 1: Table S1. The target genes used in the gene expression studies.
