# Supplementary material for: Enhanced xylose fermentation and ethanol production by engineered Saccharomyces cerevisiae strain
Source: AMB Express. 2015 Feb 26;5:16. doi: 10.1186/s13568-015-0102-y (PMC4385029; doi:10.1186/s13568-015-0102-y)
Supplement: Additional file 1: Table S1. — The target genes used in the gene expression studies. [file 13568_2015_102_MOESM1_ESM.pdf]

Supplementary material

Enhanced xylose fermentation and ethanol production by engineered *Saccharomyces cerevisiae* strain

Leonardo de Figueiredo Vilela[1]; Verônica Parente Gomes de Araujo[1]; Raquel de Sousa Paredes[1];Elba Pinto da Silva Bon[1]; Fernando Araripe Torres[2]; Bianca Cruz Neves[1]; Elis Cristina Araújo Eleutherio[1]*

[1]Department of Biochemistry, Institute of Chemistry, Federal University of Rio de Janeiro, Rio de Janeiro, Brazil

[2]Department of Cellular Biology, Institute of Biology, University of Brasília, Brasília, DF Brazil

*Corresponding author

Address:

Av. Athos da Silveira Ramos 149, C.T., Bl. A, lab 547

CEP: 21941-909, Rio de Janeiro, RJ, Brazil

Phone/FAX: +55 21 2562-7735/7826

E-mail: eliscael@iq.ufrj.br

Table S1 - The target genes used in the gene expression studies

**Gene**	**Protein**	**Function***
XKS1	Xylulokinase	Converts D-xylulose and ATP to xylulose 5-phosphate and ADP
RPE1	D-ribulose-5-phosphate 3-epimerase	Converts D-ribulose 5-phosphate to D-xylulose 5-phosphate
RKI1	Ribose-5-phosphate ketol-isomerase	Catalyzes the interconversion of ribose 5-phosphate and ribulose 5-phosphate
TAL1	Transaldolase	Converts sedoheptulose 7-phosphate and glyceraldehyde 3-phosphate to erythrose 4-phosphate and fructose 6-phosphate
TKL1	Transketolase	Catalyzes conversion of xylulose-5-phosphate and ribose-5-phosphate to sedoheptulose-7-phosphate and glyceraldehyde-3-phosphate
HXT1	Hxt1p	Low-affinity glucose transporter
HXT2	Hxt2p	Intermediate/Low-affinity glucose transporter
HXT7	Hxt7p	High-affinity glucose transporter

*Ref: Cai et al. 2012
